# 문제중심학습 기반 가상현실 시뮬레이션 교육이 간호대학생의 비판적 사고능력, 문제해결능력 및 자기효능감에 미치는 효과: 유사실험 연구

**DOI:** 10.4069/kjwhn.2023.09.12

**Published:** 2023-09-26

**Authors:** Young A Song, Minkyeong Kim

**Affiliations:** Department of Nursing, Ansan University, Ansan, Korea; 안산대학교 간호학과

**Keywords:** Problem-based learning, Problem solving, Self efficacy, Simulation, Virtual reality, 문제중심학습, 문제해결, 자기효능감, 시뮬레이션, 가상현실

## Abstract

**Purpose:**

This study analyzed the effects of virtual reality simulation-based problem-based learning on nursing students’ critical thinking ability, problem-solving ability, and self-efficacy in the nursing care of women undergoing induction of labor.

**Methods:**

A nonequivalent control group pretest and posttest design was employed. The study participants included 52 nursing students (24 in the experimental group and 28 in the control group). The experimental group took a problem-based learning (PBL) class in the first week, and then engaged in self-directed learning using virtual reality simulation. In the second week, lectures about emergency nursing care for induction of labor and drug administration were given. The control group participated in PBL in the first week and lectures in the second week. The study was conducted from April 17 to May 19, 2023. Data were analyzed using the chi-square test, Fisher exact test, analysis of variance, and the independent t-test.

**Results:**

Before-and-after differences between the two groups were statistically significant in problem solving ability (t=–5.47, *p*<.001) and self-efficacy (t=–5.87, *p*<.001). Critical thinking ability did not show a statistically significant difference between the two groups. The score for satisfaction with the virtual reality simulation program was 3.64±5.88 out of 5 in the experimental group.

**Conclusion:**

PBL education using a virtual reality simulation was found to be an effective way of teaching. Although convenience sampling was used, PBL education using virtual reality can be used as an educational strategy to enhance nursing students’ problem-solving ability and self-efficacy.

## Introduction

현대 사회가 빅데이터 시대로 전환되면서 정보는 중요한 자원으로 대두하였다. 교육 분야에서도 막대한 정보량으로 교수-학습자의 역할 변화가 요구되고 있다. 지식 전달에 초점을 맞추던 암기 위주의 교육에서, 수많은 정보 중에서 학습자가 스스로 필요한 데이터를 파악하고 선택하여 문제를 해결해야 하는 형태로 변화되었기 때문이다. 이에 대학은 통합적 사고력을 갖춘 창의적 인재를 육성하기 위해 학습자를 고려하여 다양한 교수법을 연구하고 있다[1].

이런 관점에서 대학은 교수가 주도하는 전통적 강의형식을 탈피하고 학습자 중심의 교육활동을 강조하고 있다. 플립드러닝(flipped learning), 블렌디드 러닝(blended learning), 문제중심학습법(problem-based learning), 팀 러닝(team-based learning, TBL), 프로젝트 기반 학습 등이 학습자 중심의 교수학습방법의 예이다[1-3].

문제중심학습은 많은 연구에서 긍정적 학습 효과가 보고된 교수학습방법 중 하나이다. 문제중심학습은 지식의 습득과 이해에 초점을 두지 않고, 지식과 관련된 문제상황을 해결하는 과정을 중시한다. 따라서 학습이 발생하는 문제해결 도출과정에 주안점을 두고 수업 절차를 구성하는 것이 특징이다[4]. 교수자는 실제 임상에서 발생하는 비구조화된 문제상황을 제시하고, 학습자는 문제를 해결하기 위해 자신의 지식을 바탕으로 고민하고 동료 학습자와 의견을 나눈다. 그 과정에서 추론과 응용을 해봄으로써 지식의 확장과 전이를 경험할 수 있다. 결론적으로 학습자는 문제상황을 해결하는 과정을 통해 창의력, 자기효능감, 비판적 사고력, 문제해결능력을 향상할 수 있기 때문에, 복잡하고 다양한 임상 상황에서 의미 있는 정보를 구별하고 판단해야 하는 문제중심학습은 효과적인 학습방법으로 주목받고 있다[5,6].

간호사는 단순질환보다 복합적 건강문제가 많은 임상현장에서 근무하므로 대상자의 상황에 대하여 비판적으로 사고하여 문제를 해결해야 한다. 특히 대상자의 상태가 갑자기 변화하는 경우, 간호사는 그 상황에 맞는 지식을 통합적으로 적용하여 빠르고 논리적인 의사결정을 해야 한다. 문제중심학습은 학습 내용에 대한 심층적 이해를 도울 뿐만 아니라 간호문제로 학습을 전이하여 문제해결력의 향상을 꾀할 수 있으므로 간호학 교육에 적절한 교수법이라 할 수 있다[7,8].

하지만 학습자가 주어진 문제를 해결하려는 목적의식이 있을지라도 비판적 사고력을 갖추지 못했거나, 문제중심학습이 학습자에게 부담으로 작용한다면 실질적인 학습 효과를 기대하기는 어렵다[9]. Kim 등[10]은 문제중심학습에서 자기효능감을 효과적으로 증진하려면 학습자가 교육에 참여하는 활동도 중요하지만, 편안한 환경에서 자신의 의지로 반복하며 학업을 수행할 수 있는 과정이 필요하다고 강조하였다. 따라서 학습자가 자신에 맞추어 학습 속도를 조절하고 능동적으로 참여하여 최적의 학습 효과를 창출할 수 있도록 문제중심학습을 설계해야 한다.

가상현실 시뮬레이션(virtual reality simulation)은 4차 산업 확대로 교육현장에 도입된 온라인 콘텐츠이다. 이는 임상 현장을 기반으로 한 시나리오로 구성되며, 각 상황에 대한 임상적 판단을 통해 문제해결능력의 함양을 기대한다[11,12]. 가상현실 시뮬레이션이 학습 실재감이 있고 공간 제약은 없다는 장점 때문에, COVID-19 범유행 상황이 발생한 후 많은 대학이 교육목표 달성을 위해 실습의 대체 교육으로 활용하고 있다. 간호교육에서는 성인간호학실습, 아동간호학실습 등 대부분 실습 교과목에서 가상현실 시뮬레이션을 적용하고 있다[13]. 국내 연구를 살펴보면 가상현실 시뮬레이션을 활용한 실습교육은 학습 속도의 유연성을 높이고 반복 수행이 가능하여 학습자의 지식과 실습만족도를 향상시키는 것으로 보고된다[14]. 또한 가상현실 시뮬레이션은 임상수행능력, 간호수행자신감, 그리고 문제해결능력의 일부 하위 영역을 향상하는 효과가 있었다[11]. 이러한 점은 가상현실 시뮬레이션을 간호학실습에서 이론수업으로 확대 적용할 가능성을 시사한다. 이미 언급한 바와 같이 비판적 사고가 요구되는 이론 수업에서 학습 내용을 깊이 있게 이해하기 위해 문제중심학습을 도입했다 하더라도 학습자가 적은 부담으로 스스로, 반복적으로 접근 가능할 때 학습 효과가 나타난다. 이때 가상현실 시뮬레이션을 함께 적용한다면 문제중심학습을 보완하는 긍정적 효과를 얻을 수 있을 것이다. 두 가지 교수법의 시너지 효과를 창출하려면 가상현실 시뮬레이션이 문제해결과정 중 탐색 과정, 중재 선택, 선택한 결과의 확인과 평가가 가능하다는 장점을 활용하여[11] 수업을 구성할 때 학습자에게 다양한 해결방안을 고민하고 적용해볼 수 있는 기회를 제공하도록 순서를 고려해야 한다. 가상현실 시뮬레이션과 문제중심학습을 융합한 이론수업은 COVID-19 범유행 중에 온라인 콘텐츠 학습을 경험하고 디지털 사용에 친숙한 현 세대의 특성을 생각할 때 문제중심수업 효과보다 더 높은 학습 효과를 보여줄 것이다[9]. 이러한 점에 착안하여 다양한 지식을 통합한 사고력을 요구하는 간호교육에서 비판적 사고를 촉진하고 자신감을 향상할 수 있도록 가상현실 시뮬레이션 자기주도학습과 문제중심학습법을 결합한 교수법을 구상하게 되었다.

우리 사회는 만혼과 고령 임신이 증가하면서 임신과 분만 합병증 발생률이 증가하고 이에 따라 유도분만 역시 계속 증가하고 있다. 유도분만 상황은 산부와 태아 상태에 대한 통합적 이해와 관찰이 요구되므로 간호사의 정확한 지식, 비판적 사고와 문제해결능력이 매우 중요하다. 그러나 사생활 보호 요청과 출산율 저하로 분만실 임상실습 참여조차 점점 더 어려워지고 있다.

이에 본 연구에서는 간호 학생이 유도분만 상황을 정확히 이해하고 다양한 지식을 통합하여 비판적으로 사고할 수 있도록, 유도분만에 대한 문제중심학습과 가상현실 시뮬레이션 교육을 융합한 교수법을 설계하고 그 효과를 파악하고자 한다.

본 연구의 목적은 유도분만 산부 간호에서 문제중심학습 기반 가상현실 시뮬레이션 교육이 간호대학생의 비판적 사고능력, 문제해결능력 및 자기효능감에 미치는 효과를 검증하고 학습자의 가상현실 시뮬레이션 교육만족도를 확인하기 위함이다.

연구 가설은 다음과 같다.

(1) 가설 1. 문제중심학습 기반 가상현실 시뮬레이션 교육에 참여한 실험군은 일반 문제중심학습에 참여한 대조군과 비판적 사고성향 점수에 차이가 있을 것이다.

(2) 가설 2. 문제중심학습 기반 가상현실 시뮬레이션 교육에 참여한 실험군은 일반 문제중심학습에 참여한 대조군과 문제해결능력 점수에 차이가 있을 것이다.

(3) 가설 3. 문제중심학습 기반 가상현실 시뮬레이션 교육에 참여한 실험군은 일반 문제중심학습에 참여한 대조군과 자기효능감 점수에 차이가 있을 것이다.

## Methods

Ethics statement: Obtaining informed consent was exempted by the Institutional Review Board of Ansan University (2023-04-001) because there was no sensitive information and the survey was anonymously treated. To encourage voluntary participation a professor independent to the research team explained the study purpose, principles of confidentiality, right to withdraw, and ensured that participation was not related to grading. The program was offered to comparison group students after completion of the study (28 students participated).

### 연구 설계

**Figure 1.**
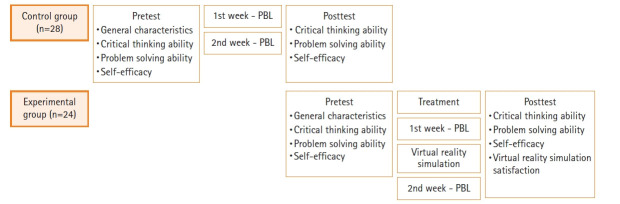
Research design. PBL: problem-based learning.

본 연구는 유도분만 상황 시나리오의 문제중심학습 기반 가상현실 시뮬레이션을 경험한 실험군과 일반 문제중심학습에 참여한 대조군의 비판적 사고성향, 문제해결능력, 자기효능감의 차이를 검증하고, 가상현실 시뮬레이션 교육만족도를 확인하기 위한 비동등성 대조군 전후 시차설계(nonequivalent control group non-synchronized design)를 적용한 유사실험 연구이다(Figure 1).

### 연구대상 및 표집

본 연구는 경기도 소재의 간호학과 학생을 대상으로 다음 선정기준을 적용하여 편의표집하였다. 구체적 기준은 다음과 같다.

(1) 본 연구의 목적을 이해하고 자발적으로 동의서에 서명한 자

(2) 간호학과 교과목 여성건강간호학 I 이수자

(3) 가상현실 시뮬레이션 실습교육 무경험자

표본 크기는 Cohen [15]이 제시한 표를 근거로 설정하였고, G-power 3.1.2 프로그램을 활용하였다. Yang과 Hong [16]의 연구에 근거하여 효과크기(d)=.80, 검정력(1–β)=.80, 유의수준(α)=.05으로 분석하였을 때 각 군에 26명씩 산출되었다. You와 Yang [11]의 중도탈락률(10%)을 고려하여 각 군에 30명씩 배정하였다.

**Figure 2.**
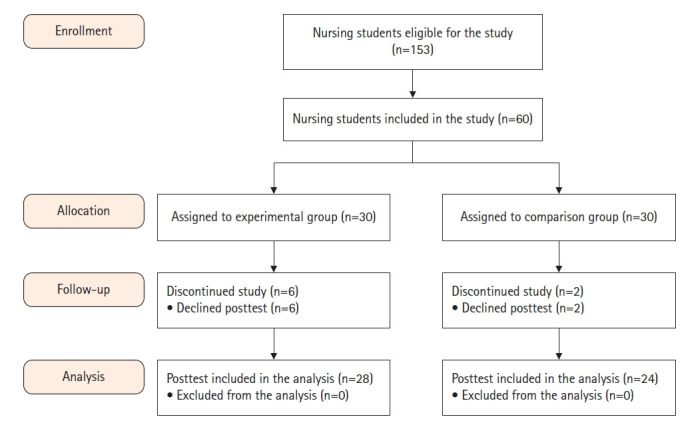
Flow diagram in this study.

실험군 중 COVID-19 감염 결석(n=2)과 개인 사정(n=2)으로 2주차 대면 수업에 참여하지 못했으며, 가상현실 시뮬레이션에 참여하지 않은 2명을 포함하여 총 6명의 탈락으로 최종 24명이 분석에 포함되었다. 대조군은 COVID-19 감염으로 2주차 대면 수업에 참여하지 못한 2명을 제외한 최종 28명이 분석에 포함되었다(Figure 2).

### 연구 도구

#### 비판적 사고성향

비판적 사고성향은 Yoon [17]이 개발한 비판적 사고성향 측정도구를 사용하였다. 본 도구는 7개의 하위영역으로 구분하여, 지적열정/호기심 5문항, 신중성 4문항, 자신감 4문항, 체계성 3문항, 지적 공정성 4문항, 건전한 회의성 4문항, 객관성 3문항 등 총 27문항으로 구성되며, 각 문항은 ‘전혀 그렇지 않다’ 1점에서 ‘매우 그렇다’ 5점의 Likert 척도로, 점수가 높을수록 비판적 사고성향이 높음을 의미한다. 이 중 2문항(4, 14)은 부정문항으로 점수의 일관성을 위해 역환산 처리하였다. 도구 개발 당시 신뢰도는 Cronbach’s α 계수는 .84이고 본 연구에서 Cronbach’s α는 .83이었다.

#### 문제해결능력

문제해결능력은 한국교육개발원에서 Lee 등[18]이 개발한 문제해결능력 진단지를 사용하였다. 이 도구는 총 45문항으로 각 문항은 5점 Likert 척도로 측정되며 점수가 높을수록 임상수행능력이 높음을 의미한다. 이 중 2문항(8, 10)은 부정문항으로 점수의 일관성을 위해 역환산 처리하였다. 도구 개발 당시 신뢰도는 Cronbach’s α 계수는 .84, Lee 등[18]의 연구에서의 Cronbach’s α는 .94이었으며, 본 연구에서 Cronbach’s α는 .88이었다.

#### 자기효능감

자기효능감 측정을 위해 19.DiIorio와 Price [19]가 개발한 ‘Neuroscience Nursing Self-efficacy Scale’을 Kim [20]이 수정·보완한 자기효능감 도구를 본 연구자가 유도분만간호 사례에 맞게 수정하였다. 이 도구는 총 17문항으로, ‘전혀 잘 할 수 없다’ 0점에서 ‘아주 확실히 할 수 있다’ 10점의 11점 Likert 척도로 0–170점의 범위를 갖는다. 점수가 높을수록 자기효능감이 높음을 의미한다. Kim [20]의 연구에서 도구의 신뢰도 Cronbach’s α는 .95였고 본 연구의 Cronbach’s α는 .96이었다.

#### 가상현실 시뮬레이션 교육만족도

가상현실 시뮬레이션 교육만족도를 측정하기 위하여 Oh와 Kim [21]이 개발한 10문항을 원저자의 허락을 받아 수정·보완하여 사용하였다. 프로그램 진행의 내용, 수행 시간, 구성 등의 적절성 등에 대하여 ‘매우 그렇지 않다(1점)’에서 ‘매우 그렇다(5점)’까지의 5점 Likert 척도로 측정햐였으며, 점수가 높을수록 프로그램 교육만족도가 높음을 의미한다. Oh와 Kim [21] 연구에서 Cronbach’s alpha는 .95였으며, 본 연구에서의 Cronbach’s α는 .93이었다.

### 자료 수집

본 연구는 안산시에 소재한 1개 대학에서 여성건강간호학 Ⅱ 교과목을 수강하는 간호학과 3학년 학생 중 자발적으로 연구 참여에 동의한 자를 대상으로 시행하였다. 해당 강의에 참여하지 않는 연구자가 연구 목적과 방법, 자료 처리와 폐기, 참여하지 않을 권리, 수업 및 평가와 무관함을 충분히 설명하고 연구 참여 동의서를 받은 후 수행하였다. 연구 기간은 2023년 4월 17일부터 5월 19일까지 1개월로 웹 설문지를 활용하여 수집하였다. 해당 대학은 6개반(A–F반)으로 구분되어 있으며, 3개반씩 이론강의와 임상실습이 교차하여 진행되었다. D–F반이 임상실습을 하는 4월 24일에서 5월 5일의 2주 동안 A–C반은 학교에서 대면 수업을 진행하였다. 따라서 A–C반을 대조군으로 배정하여 일반 문제중심학습을 하였다. 이어서 A–C반이 임상실습을 하는 5월 8일에서 5월 19일의 2주 동안에는 D–F반을 실험군으로 임의 배정하여 학교에서 대면 수업으로 문제중심학습 기반 가상현실 시뮬레이션 교육을 하였다. 연구 기간 내 이론 반과 실습 반의 접점이 없어 실험 처치에 대한 오염과 확산을 최소화하였다.

설문 소요시간은 20분 정도였으며, 연구 참여자에게는 설문 완료 후 소정의 선물을 제공하였다. 대상자들은 2학년 2학기에 여성건강간호학에서 여성건강개념과 임신과 임부간호, 분만생리와 산부간호를 학습하였다.

### 연구 진행절차

#### 문제중심학습 기반 가상현실 시뮬레이션 교육 설계

대조군과 실험군 설정은 실험 처치의 확산을 예방하기 위해 비동등성 대조군 전후 시차설계를 하였다. 이론수업을 먼저 진행하는 반(A–C반)을 대조군, 임상실습을 마치고 2주 후 이론수업에 참여하는 D–F반을 실험군으로 배정하였다. 대조군과 실험군 각각 문제중심학습 시작 전에 웹 설문지를 통해 일반적 특성, 비판적 사고성향, 문제해결능력, 자기효능감을 조사하였다.

두 집단에게 동일한 유도분만 패키지를 적용하여 문제중심학습 교수법을 진행하였다. 두 군은 동일하게 조당 4–5명, 6–7개 조로 구성하였다. 대조군의 1주차 수업은 유도분만 상황에 대한 문제중심학습이다. 문제중심학습 수업에서 사용한 모듈은 가상현실 시뮬레이션 “labor induction due to gestational diabetes”를 기반으로 구성하였다. 학습자는 유도분만 시나리오를 제공받고 문제해결 접근방법에 따라 협동학습을 하며 상호 피드백을 하였다. 2주차 수업은 ‘더 알고 싶은 것(learning issues)’을 동료와 함께 학습하며 학습 결과를 공유하고, 조별 성찰저널(reflective journal)을 작성하고 제출하도록 하였다. 수업은 유도분만에 대한 교수자의 강의와 조별 발표에 대한 교수자의 피드백으로 마무리하였다.

실험군의 1주차 수업은 대조군과 동일하게 진행하였으며, 1주차 수업 종료 후 가상현실 시뮬레이션 프로그램에 대한 내용과 절차를 e-Class에 공지하였다. 본 연구에서 적용한 가상현실 시뮬레이션 프로그램은 Laerdal Medical Korea 2020의 vSim for Nursing이다. 각 시나리오는 ‘Core’와 ‘Complex’로 구분되고, 시나리오마다 6단계로 구성되어 있다. 1단계는 ‘suggested reading’으로 학습 목표, 학습 내용, 대상자 정보가 제시되고, 2단계 ‘pre-simulation quiz’에서 vSim 사례에 대한 퀴즈를 통해 지식을 평가하며 정답과 해설을 제공한다. 3단계 vSim에서 학습자는 가상현실 시뮬레이션 환경에서 간호사정, 대상자 교육, 의사소통, 간호중재 선택, 의사지시 확인 등 간호를 수행한다. 학습자가 수행한 결과는 백분율(%)과 함께 피드백을 제공받을 수 있다. 시뮬레이션 종료 후 4단계 ‘post-simulation quiz’에서 vSim을 통해 얻은 지식을 재확인한다. 5단계 ‘document assignments’는 학습에 도움이 되는 과제물이 있으며, 6단계 ‘guide reflection question’은 시뮬레이션 과정에 대한 성찰 질문이 제시된다. 본 연구에서는 모성간호영역(vSim for Nursing Maternity) 중 oxytocin을 사용한 유도분만간호 “labor induction due to gestational diabetes” 모듈을 선택하였다.

실험군은 가상현실 시뮬레이션을 2주차 수업 전까지 완료하도록 하였다. 수료 기준은 100점 만점에 80점 이상으로 제시하였으며, 평균 3.5회(최소 2회–최대 5회) 반복학습을 수행하였다. 2주차 수업은 대조군과 동일하게 진행하였다.

총 2주간의 중재가 종료된 후, 각각 대조군과 실험군에게 웹 설문지를 통해 비판적 사고성향, 문제해결능력, 자기효능감을 조사하였으며, 실험군은 가상현실 시뮬레이션 교육만족도를 추가 조사하였다.

### 자료 분석

수집된 자료 분석은 IBM SPSS ver. 23.0 (IBM Corp., Armonk, NY, USA)을 이용하여 분석하였다. 정규분포 유무를 확인하기 위해 Kolmogorov-Smirnov test를 실시하였다. 대상자의 일반적 특성과 각 변수의 값은 빈도, 백분율, 평균과 표준편차로 분석하였으며, 두 집단 간 동질성 검정은 카이제곱 검정, Fisher 정확 검정, 독립 t-검정, 분산분석으로 분석하였다. 두 집단 간 차이 검정은 독립 t-검정을 이용하여 분석하였다. 측정도구의 신뢰도는 Cronbach’s coefficients ⍺로 산출하였다

## Results

### 대상자의 일반적 특성과 사전 동질성 검정

실험군과 대조군의 일반적 특성과 종속변수에 대한 사전 동질성 검정은 다음과 같다(Table 1). 두 집단의 평균 연령, 성별, 간호학과 입학 동기, 선호하는 학습방법, 대인관계 만족도는 실험군과 대조군 간에 유의한 차이가 없어 두 군의 일반적 특성은 동질한 것으로 나타났다(Table 1). 측정변수의 사전 동질성을 검증한 결과, 두 집단의 비판적 사고성향, 문제해결능력, 자기효능감 점수는 통계적으로 유의하지 않아 동질한 특성을 가진 그룹으로 나타났다(Table 1).

### 문제중심학습 기반 가상현실 시뮬레이션 교육의 효과 검정

실험군의 비판적 사고성향은 사전 91.21±9.15점, 사후 103.75±10.18점으로 총 9.50±9.33점이 상승했으며, 대조군의 비판적 사고성향은 사전 94.25±7.02점에서 사후 99.11±12.33점으로 6.89±8.41점 상승하였다. 그러나 비판적 사고성향의 두 그룹 간 사전-사후 차이는 통계적으로 유의하지 않아(t=–1.47, *p*=.149) 제1가설은 지지되지 않았다.

문제해결능력은 실험군이 사전 152.50±11.39점, 사후 192.75±14.85점으로 40.25±18.18점 상승하였고, 대조군의 문제해결능력은 사전 152.93±12.42점, 사후 167.96±17.43점으로 15.04±15.97점 상승하였다. 두 그룹 간 사전-사후 차이를 검증한 결과 실험군이 대조군보다 통계적으로 유의하게 문제해결능력이 상승한 것으로 나타나(t=–5.14, *p*<.001) 제2가설은 지지되었다.

자기효능감은 실험군이 사전 97.88±19.63점, 사후 142.00±13.91점으로 44.13±23.82점 상승하였고, 대조군은 사전 94.93±25.14점, 사후 112.36±21.10점으로 17.43±23.36점 상승하였다. 자기효능감의 두 그룹 간 사전-사후 차이를 검증한 결과 실험군이 대조군보다 유의하게 상승한 것으로 나타나(t=–5.87, *p*<.001) 제3가설은 지지되었다(Table 2).

### 가상현실 시뮬레이션 교육만족도

가상현실 시뮬레이션(vSim for Nursing)을 경험한 후 실험군의 가상현실 시뮬레이션 교육만족도는 Table 3과 같다. 가상현실 시뮬레이션 프로그램에 대한 교육만족도는 5점 만점 중 3.64±5.88점이었다. 만족도가 가장 높았던 항목은 유도분만간호에 대한 관심도 상승(3.81±0.79점)이었으며, 다음으로 반복학습의 유용성(3.79±0.78점)과 가상현실 시뮬레이션 화면의 구성충실도(3.75±0.62점) 순으로 높았다. 반면 본 프로그램이 영어로 구현되기 때문에 사용언어(3.42±0.80점)와 이해도(3.42±0.80점)에 대한 만족감이 가장 낮았고, 가상현실 시뮬레이션 프로그램 사용에 대한 설명과 수행(3.54±0.83점)에서 만족도가 낮았다.

## Discussion

유도분만은 산모와 태아 상태에 대한 동시 판단이 필요하며 약물 부작용과 제왕절개분만 상황처럼 예상치 못한 상황을 염두에 두어야 한다. 특별한 문제가 발생하지 않는다 하더라도 분만 진행에 따라 산모의 불안과 진통이 증가하므로 통합적인 간호가 필요하다. 이에 본 연구는 간호대학생이 유도분만간호에 대해 정확한 지식을 기반으로 빠른 상황 판단과 정보의 선택, 그리고 문제해결능력의 함양을 위해 학습자가 능동적으로 참여하고 상호작용할 수 있는 문제중심학습 기반 가상현실 시뮬레이션 교육을 간호대학생에게 적용한 결과를 토대로 논의하고자 한다.

본 연구에서 문제중심학습 후 가상현실 시뮬레이션 교육을 적용한 실험군이 문제중심학습만을 적용한 대조군보다 유의하게 향상된 변수는 문제해결능력과 자기효능감이다. 문제해결능력에 대한 연구 결과는 간호대학생에게 가상현실 시뮬레이션 프로그램으로 성인간호 시나리오를 제공했을 때 문제해결능력이 상승한 You와 Yang [11]의 연구 결과와 유사하였으며, 천식아동간호에 대한 가상현실 시뮬레이션의 실습교육 효과를 확인한 연구에서 문제해결능력 점수가 유의하게 상승하였다는 보고와도 유사하다[22]. 가상현실 시뮬레이션의 큰 장점은 반복적 학습이 가능하고 학습 실재감이 있다는 것이다. 그러나 가상 공간에서 학습이 진행되므로 사전 지식이 없다면 학습자 간 격차가 학습의 장애요인이 될 수 있다. You와 Yang [11]은 실험군에게 5개의 성인간호학 질환 시나리오를 제공하며 지속적으로 질의응답을 격려하였고, Kim 등[22]은 천식아동간호 가상현실 시뮬레이션을 진행하기 전 천식아동간호에 대한 강의를 통해 이론에 대한 학습을 제공하였다. 두 연구 모두 문제중심학습을 사전에 채택하지는 않았으나 가상현실 시뮬레이션의 한계점을 인지하고 최소한의 보완책을 마련한 것으로 볼 수 있다. 이 점을 고려하여 연구팀은 교수법 구성 시 문제중심학습을 사전에 실행하여 어려움은 동료와 함께 해결하여 학습의 부담감은 줄이고 비판적 사고를 촉진하도록 돕고, 이후 가상현실 시뮬레이션을 배치하여 학습자가 반복적으로 속도를 조절하며 자기주도학습을 통해 문제를 해결하도록 하였다. 학생들은 본 연구에서 제시한 가상현실 시뮬레이션(vSim for Nursing) 수료 점수인 최소 80점 이상에 달성할 때까지 최소 2번부터 최대 5번까지 평균 3.5회의 학습을 수행하였다. Kim [23]은 웹 기반 선행학습 횟수가 증가할수록 간호에 대한 자기주도적 학습능력이 높아진다고 하였다. 따라서 신속하고 정확한 판단이 요구되는 상황을 실재감이 있는 가상현실 시뮬레이션 시나리오에 적용하는 것은 가상의 임상상황을 스스로 반복하며 경험함으로써 문제해결능력을 향상하는 데 효과적이라 할 수 있다[11].

그러나 융합적 교수법이 모두 효과적인 것은 아니다. 온라인 간호술기 프로그램인 ‘Nursing Skills’를 활용하여 간호대학생의 기본간호학 실습에서 블렌디드 러닝을 적용한 Jho [24]의 연구에서는, 메타 인지는 유의하게 증가하였으나 문제해결능력은 교육 전후 차이가 나타나지 않았다. 온라인 간호술기 프로그램은 가상현실 프로그램이지만 시나리오로 구현되지 않기 때문에 학습 실재감이 낮을 수 있으며, 단순 술기로 암기에는 도움이 될 수 있으나 상위 개념인 문제해결능력을 촉진하기는 어려울 수 있다[24]. Jho [24]의 연구에서 학습자는 매주 온라인으로 자가학습을 한 후 교수자 강의에 참여하였다. 만일 학습자의 선수지식이 부족하면 온라인에서 학습 점수가 낮고, 이로 인해 학습동기가 생기지 않아 문제해결능력에 영향을 미칠 것으로 생각된다. 이러한 점을 고려하면, 여성건강간호학에서 문제중심학습 기반 가상현실 시뮬레이션 교수법은 유도분만과 같이 모성-태아 상황을 다각적으로 판단해야 하거나 복잡한 간호문제 학습에서 문제해결능력을 향상할 수 있는 매우 효과적인 방법이라 볼 수 있겠다.

자기효능감은 ‘어떤 상황에서 적절한 행동을 할 수 있다는 기대와 신념’, 즉 ‘과제를 수행하는 데 있어 자신이 그 과제를 성공적으로 수행할 수 있을 거라는 능력에 관한 신념’을 의미한다[25]. 본 연구에서 문제중심학습 후 가상현실 시뮬레이션 교육 후 자기효능감의 효과에 대한 결과는 가상현실 시뮬레이션 교육이 간호대학생의 자기효능감에 긍정적이라는 기존 연구 결과에 부합하는 결과이다[25]. 그러나 본 연구 결과와 다르게 가상현실을 활용한 수업에 참여한 실험군과 강의식 수업에 참여한 대조군 간의 자기효능감은 유의한 차이를 보이지 않았다는 보고도 있다[12]. Kim과 Kim [12]의 연구는 가상현실을 활용하여 병원 환경 지식을 학습하는 기본간호학 수업으로 대상자들의 지식은 향상되었으나 병원 물품이나 위치, 기구 확인 위주로 과제의 난이도가 낮았으므로 학업적 자기효능감이 낮게 나타난 것으로 생각할 수 있다[12]. 자기효능감은 단방향의 학습보다 학습 실재감이 있는 대리경험이나 실제적 수행경험, 상호작용을 통한 칭찬과 격려로 형성될 수 있다[12]. 자신감이 높을수록 자기효능감 역시 높아진다[26]. You와 Yang [11]은 가상현실 시뮬레이션을 적용한 후 간호수행자신감을 비교하였는데, 실험군과 대조군 간에 교육 전후 차이가 유의한 것으로 나타났다. Kim [27]은 자기조절학습전략과 자기효능감은 상관관계가 있어 자기조절능력이 높은 학습자일수록 학습과정에 고도의 자기효능감을 보여줄 뿐 아니라 괄목한 만한 수준의 성과를 달성한다고 하였다. 가상현실 시뮬레이션 프로그램은 학습 과정에서 학습자 스스로 컴퓨터를 클릭하며 직접 중재를 선택하고 확인하는 작업이 필요하며, 피드백 확인 후 반복학습이 가능하다. 또한 과정에 대한 평가를 시각으로 확인할 수 있어 학습자가 노력하면서 점수가 상승할 수 있다는 자신감을 갖도록 하며 주어진 상황에 익숙해지면서 자기효능감이 높아졌을 것으로 생각된다. 가상현실 시뮬레이션은 자신을 포함한 환경을 조절하고 성취 목표를 위해 피드백을 관리하는 자기조절학습을 가능하게 한다. 변화가 빠른 의학 기술에 적응하고 최신 정보를 학습하며 현장에 적용하기 위해서 자기조절학습은 매우 중요한 요인이다[28]. 그러므로 학습자들이 자기조절학습을 통해 자기효능감을 높일 수 있는 교수 설계가 이루어져야 할 것이다.

본 연구에서 비판적 사고성향은 실험군과 대조군 간의 점수 차이가 통계적으로 유의하지 않았다. 기존 연구에서 간호대학생을 대상으로 정상 신생아간호에 대한 블렌디드 시뮬레이션 교육에서 가상현실 시뮬레이션을 적용한 후 비판적 사고성향에서 유의한 차이를 보고한 것과는 차이가 있다[29]. 정상 신생아간호에 대한 블렌디드 교육은 가상현실 시뮬레이션과 고충실도 시뮬레이션으로 본 연구의 중재와는 다소 차이가 있어 직접적 비교는 어렵다. 본 연구에서 비판적 사고성향이 두 그룹 간 유의한 차이를 보이지 않았던 이유는 두 그룹에게 제공된 문제중심학습 수업이 임상 시나리오 상황에서 중요한 의미를 찾고 분석하며, 간호문제 해결을 위해 접근하면서 더 학습해야 할 것(learning issue)을 찾아 자기주도학습을 하도록 촉진한 결과로 해석할 수 있다.

가상현실 시뮬레이션 교육만족도는 5점 만점에 3.64점이었다. 단일군 대상 가상현실 시뮬레이션을 적용한 실습만족도에서 교육 전 5점 만점에 4.18점에서 교육 후 4.47점으로 통계적으로 유의하게 높게 보고하였으며[30], 병원 환경에서 가상현실을 활용한 수업에 참여한 실험군의 교육만족도는 5점 만점에 4.80점, 강의식 수업에 참여한 대조군은 4.10점으로 본 연구 결과보다 높았다[12]. 단일군을 대상으로 한 연구는 가상현실 시뮬레이션을 적용하였으나 시뮬레이션 프로그램이 아닌 실습에 대한 전반적 만족도 조사로 본 연구 결과와 비교는 어렵다[30]. Kim과 Kim [12]의 연구에서 사용한 자료는 8개의 병원 공간과 물품, 기자재를 가상현실 영상으로 제작하여 시각적 이해를 돕고 한국어로 되어 있어 쉽게 이해할 수 있는 자료인 반면, 본 연구에서 사용한 가상현실 시뮬레이션은 미국에서 제작되어 영어로 구현되고 스토리를 전반적으로 이해하며 학습해야 하는 어려움이 있어 본 연구의 교육만족도가 낮게 나타난 이유라고 추측된다. 그러므로 국내 임상 상황을 기반으로 한국어로 제작된 가상현실 시뮬레이션 개발이 필요하다.

COVID-19는 교육의 대전환을 가져왔다. 예상하지 못한 전염병의 대비뿐 아니라 사회변화 속도에 맞추어 교육 역시 변화해야 한다. 현재 간호교육에서 가상현실 시뮬레이션 교육은 실습 교과목에 주로 활용되고 있지만, 본 연구에서는 이론 교과목에서 문제중심학습법과 연계하여 학습자의 자율성을 고려하여 적용할 수 있는 하나의 방안으로 제시하였다.

본 연구에서 적용한 문제중심학습 기반 가상현실 시뮬레이션 교육의 의의는 첫째, 실습 교과목 위주로 적용하던 가상현실 시뮬레이션을 간호학 수업에 적용하고 문제해결능력과 자기효능감 효과를 검증했다는 점이다. 이는 실습과 이론 교육의 통합적 관점을 학습자에게 제공할 수 있다는 면에서 효과적일 것으로 기대한다. 둘째, 대상자와의 상호작용이 중요한 분만과정을 포함한 시뮬레이션 교육에서 학습자 중심의 융합 교육방법을 제시했다는 의의가 있다. 셋째, 본 연구 결과를 바탕으로 문제 상황에 직면하였을 때 통합적 사고가 요구되는 분만간호 교육에 융합적 교수법을 적용함으로써 학습자의 문제해결능력과 자기효능감을 고취시키는 데 도움을 줄 수 있다.

본 연구는 문제중심학습 후 시나리오 상황과 연계한 가상현실 시뮬레이션 자기주도 반복학습을 하도록 한 결과, 문제중심학습과 가상현실 시뮬레이션 융합교육이 학생들의 문제해결능력과 자기효능감 향상에 효과적인 것을 확인하였으나 몇 가지 제한점이 있다. 본 연구가 일개 교육기관의 간호대학생으로 국한되었음을 고려할 때 연구 결과를 일반화할 수 없다. 또한 실습 교과목에 주로 활용되던 가상현실 시뮬레이션 교육을 이론 교과목에 적용하기 위해 단일 주제로 2주간 진행하고 중재 효과를 1회 측정하여 시간 경과에 따른 효과를 지속적으로 확인하지는 못했다. 이러한 제한점을 바탕으로 유도분만간호 외의 범위로 확대 적용하여 반복 측정할 것을 제언하며, 여성건강간호학에서 다빈도 질환의 문제중심학습 모듈을 개발하고, 모듈과 연계된 가상현실 시뮬레이션 교육 프로그램을 개발하여 수업에 활용할 수 있는 교수법 설계가 필요함을 제언한다.

## Figures and Tables

**Table 1.** Homogeneity of general characteristics and dependent variables between the experimental and control groups (N=52)

Characteristic	Categories	n (%) or M ± SD		χ^2^ or t or F(*p*)
		Experimental group (n=24)	Control group (n=28)	
Age (year)^†^		22.7±1.92	22.71±1.88	–0.22 (.826)
	21–25	22 (91.7)	24 (85.7)	0.45 (.674)
	26–28	2 (8.3)	4 (14.3)	
Sex^†^	Male	3 (12.5)	6 (21.4)	.720 (.480)
	Female	21 (87.5)	22 (78.6)	
Grade point average^†^	4.0–4.5	8 (33.3)	5 (17.9)	2.35 (.325)
	3.0–3.9	9 (37.5)	16 (57.1)	
	2.0–2.9	7 (29.2)	7 (25.0)	
Motivation for choosing nursing major^†^	Employment	10 (42.3)	12 (42.9)	2.14 (.570)
	Recommendation from others	4 (16.7)	8 (28.6)	
	Aptitude	8 (33.3)	5 (17.9)	
Preferred learning method^†^	High school grades	2 (8.3)	3 (10.7)	
	Lecture	13 (54.2)	22 (78.6)	6.65 (.132)
	Discussion	4 (16.7)	0 (0.0)	
	Self-study	3 (12.5)	2 (7.1)	
	Problem-based learning	3 (12.5)	2 (7.1)	
	Practical class	1 (4.2)	2 (7.1)	
Interpersonal satisfaction^†^	Very high	3 (12.5)	3 (10.7)	1.63 (.734)
	High	10 (41.7)	8 (28.6)	
	Ordinary	9 (37.5)	15 (53.6)	
	Low	2 (8.3)	2 (7.1)	
Critical thinking ability		92.21±9.15	94.25±7.02	–0.89 (.379)
Problem solving ability		152.50±11.39	152.93±12.42	0.13 (.897)
Self-efficacy		97.88±19.63	94.93±25.14	–0.47 (.664)

† Fisher exact test.

**Table 2.** Differences in dependent variables between groups (N=52)

Variable	Group	Mean±SD			t	*p*
		Pretest	Posttest	Difference		
Critical thinking ability	Experimental	92.21±9.15	103.75±10.18	9.50±9.33	–1.47	.149
	Control	94.25±7.02	99.11±12.33	6.89±8.41		
Problem solving ability	Experimental	152.50±11.39	192.75±14.85	40.25±18.18	–5.47	<.001
	Control	152.93±12.42	167.96±17.43	15.04±15.97		
Self-efficacy	Experimental	97.88±19.63	142.00±13.91	44.13±23.82	–5.87	<.001
	Control	94.93±25.14	112.36±21.10	17.43±28.36		

**Table 3.** Satisfaction with the virtual reality simulation program (N=22)

Item	Mean±SD
I’ve developed an interest in induction delivery nursing.	3.81±0.79
Repeated learning with this program is helpful.	3.79±0.78
The screens used for this program (communication, prescription, nursing intervention selection, etc.) are appropriate.	3.75±0.62
The program’s pre and post quizzes are appropriate.	3.69±0.64
The learning content of this program is interesting.	3.67±0.65
It is good that this program feels like a real situation.	3.67±0.71
The learning time is appropriate.	3.62±0.80
I can understand and perform well according to the content of this educational program.	3.54±0.83
The content of this program is easy to understand.	3.42±0.80
The language used in this program is appropriate.	3.42±0.80
Total	3.64±5.88
